# Using an Instructional Design Model to Teach Medical Procedures

**DOI:** 10.1007/s40670-016-0228-9

**Published:** 2016-01-19

**Authors:** Lawrence Cheung

**Affiliations:** Department of Medicine, University of Alberta, Edmonton, AB Canada; Division of Critical Care Medicine, University of Alberta, 3-129 Clinical Sciences Building, 11350 83 Avenue, Edmonton, AB T6G 2G3 Canada

**Keywords:** Instructional design model, Gagne’s Theory of Instruction, Medical procedures, Percutaneous chest tube insertion

## Abstract

Educators are often tasked with developing courses and curricula that teach learners how to perform medical procedures. This instruction must provide an optimal, uniform learning experience for all learners. If not well designed, this instruction risks being unstructured, informal, variable amongst learners, or incomplete. This article shows how an instructional design model can help craft courses and curricula to optimize instruction in performing medical procedures. Educators can use this as a guide to developing their own course instruction.

## Introduction

When educators design courses to teach learners how to perform medical procedures, they risk providing instruction that may be informal and unstructured [1], taught by supervisors who may lack competence in the procedure themselves [2], and based on instructional methods that are not supported by the medical education literature [3]. This, in turn, can lead to learners attempting procedures with which they are unfamiliar [4] and which they may perform incorrectly [5–7].

To reduce these pitfalls, educators can use an instructional design model when designing a course or curriculum. An instructional design model helps ensure that the learning objectives are clear, the instruction and learning experiences align with the objectives, the learning activities are similar amongst the learners, and the assessments used to determine competence are appropriate [8]. It serves as a blueprint that specifies the type, amount, and order of learning events that will occur [9].

This article will show educators how an instructional design model—in this case, Gagne’s theory of instructional design—can be used to design courses to teach procedures in medicine, using percutaneous chest tube insertion as an example.

## Gagne’s Theory of Instructional Design

In Gagne’s theory of instructional design [10], developers of the lesson plan must first determine the type of outcome that the learners must achieve; then, they construct and tailor the instructional events necessary to achieve this outcome [9]. This model has been used to develop instructional plans to teach a variety of procedural [11–13] and cognitive skills [14–18]. Gagne’s theory of instructional design posits five learning outcomes and nine events of instruction.

## Gagne’s Five Learning Outcomes

Gagne proposed five types of learning outcomes, including attitudes, motor skills, memory or recall, complex or procedural knowledge, and learning strategies [19]. The latter three involve cognitive outcomes, while attitudes and motor skills involve affective and psychomotor outcomes, respectively. Complex or procedural knowledge, in turn, encompasses five subcategories including discriminations, concrete concepts, defined concepts, rules, and higher order rules or problem solving [20].

Using the example of percutaneous chest insertion, the learning outcome would be motor skills (that is, procedural technique) and memory or recall (learning the indications, contraindications, and immediate complications of the procedure).

## Gagne’s Nine Events of Instruction

Once educators have identified the learning outcomes, they must construct and organize the instructional events to achieve these learning outcomes. Gagne proposed nine events of instruction including gaining attention, informing the learner of the objectives, stimulating recall of prerequisite learning, presenting the stimulus material, providing learning guidance, eliciting the performance, providing feedback about performance correctness, assessing the performance, and enhancing retention and transfer [20].

Adopting Gagne’s nine events of instruction, we use the following instructional blueprint when teaching our learners (that is, residents in our respirology subspecialty residency program) percutaneous chest tube insertion.

### Gaining Attention

Educators first need to gain, and maintain, the learners’ attention so that the latter can focus on the requisite learning.

We use a pre-test (and subsequent post-test near the end of instruction) to grab their attention and promote participation as learners tend to view this approach favorably [21]. Relating their learning to the workplace also helps gain their attention [22], and we emphasize that learning this procedure is a practical skill required to manage patients during their training and clinical practice. This reinforcement also stimulates their intrinsic motivation and enhances their learning autonomy. Our instructors also judiciously use humor, anecdotes, and case-based examples to emphasize their teaching points as these have been shown to capture attention [23, 24].

### Informing the Learners of the Objectives

After gaining the learners’ attention, educators must state the learning objectives. Akin to what DeSilets [25] calls a “road map” showing the educational destination, learning objectives specify to the learners and instructors the skills that should be achieved and the outcomes that will be assessed [26]. Stated from the learner’s perspective, objectives should use action verbs [27, 28] that describe observable behaviors that the learners need to demonstrate [29–32].

For example, some of our objectives include “List the equipment needed for percutaneous chest tube insertion,” “Insert the introducer needle into the pleural space,” and “Insert the chest tube over the guide wire.” We also ask learners to state their learning objective(s)—that is, what they intend to learn from the session. By focusing activities on their needs, we can engage and empower them during the learning process [33].

### Stimulating Recall of Prerequisite Learning

Stimulating recall of prerequisite knowledge serves many functions. It establishes what the learners already know and reveals deficits in pre-existing knowledge that instructors must fill before further learning occurs. It helps the learners organize this pre-existing knowledge into conceptual schemas that can facilitate learning of new material [34] and activates prior knowledge to improve the information processing that will occur in the subsequent learning [35].

To do this, we review the answers to the initial pre-test that we administered while we were gaining the learners’ attention—this act of retrieving information enhances subsequent learning and recall. Our pre-test covers the anatomy of the chest wall, lungs and pleural space, appearance of a pleural effusion on ultrasound, the diseases that can cause a pleural effusion, and the diagnostic tests needed to ascertain the cause. In addition to lower order questions that test recall (for example, draw the anatomy of the chest wall, lungs, and pleural space), we use higher order questions as well. For example, given the relevant anatomy, we ask questions on the complications that can occur during the procedure and the ways to avoid them—these higher order questions can promote deep thinking and learner engagement. We also ask individuals to explain their answers to the group as this mindful use of prior knowledge facilitates the learning of the person who formulates the explanation [36].

### Presenting the Stimulus Material

Here, new information is presented to the learner. Instructors must emphasize important learning points. In the case of teaching a procedural skill, instructors should not only emphasize the actions needed to perform the procedure correctly, but also highlight the actions to avoid in order to decrease the risk of adverse events.

When teaching percutaneous chest tube insertion, we use small groups of up to five members as this has been shown to increase learning gains and learner satisfaction compared to larger groups [37]. We review the prerequisites for the procedure—this includes obtaining informed consent and ensuring availability of all the necessary equipment, space, and personnel. Then, using photographs of each step of the procedure, we explain each of these steps such as positioning the patient, localizing the effusion with ultrasound, appropriately positioning the patient for the procedure, donning the appropriate gowns, masks, and gloves, opening the sterile tray and organizing the equipment, and so on. For each step, we emphasize the correct actions to perform and the incorrect actions to avoid. Before the teaching session, we give each learner a handout outlining the procedure so that they can prepare themselves. To promote deeper learning, we encourage the learners to ask questions and we invite group members to share their own procedural tips that they have observed in the past.

### Providing Learning Guidance

Providing learning guidance involves modeling or showing the learner the correct performance. In some cases, instruction during this phase might be very similar to that provided during presentation of the stimulus material [9]. In our case of procedural learning, providing learning guidance involves a demonstration of the whole procedure—uninterrupted—from start to finish. This integrates all of the individual procedural steps that were taught during presentation of the stimulus.

We first play a demonstrational video in its entirety without interruption; then, we replay the video—pausing intermittently to give the learners the opportunity to ask questions and/or supply comments during the second viewing.

### Eliciting the Performance

To elicit the learners’ performance, they must be given a chance to practice and demonstrate the skill they are required to learn. Before eventually performing the procedure on patients, each learner is given the opportunity to perform the procedure on a manikin that emulates the chest wall anatomy with a pleural effusion. Learners can view practice with a simulator as effective as practice on real patients [38] and simulators help learners achieve a variety of performance skills without compromising patient safety [39–46].

Each of our learners takes a turn performing the procedure while the instructor and other learners observe. Organizing the learners into groups of two—with each learner taking a turn performing and critiquing the procedure—can decrease the instructor to learner ratio [47]. We let each learner practice the procedure once, while realizing that more complex procedures benefit from serial deliberate practice and feedback over several sessions [48, 49].

### Providing Feedback About Performance Correctness

Practice by itself, without feedback, does not necessarily improve performance as learners may be unable to accurately assess themselves and determine the improvements they need to make [50–54]. And, feedback, without the learner’s reflection on how to incorporate that feedback, will not likely lead to improvement either [55]. Thus, coaching and feedback, coupled with the learner’s self-appraisal, is needed to improve performance [56–59] and enhance future self-assessment ability [60]. This is especially true when the verbal feedback is given by an expert instructor already proficient with the procedural technique [61] and is tailored to the learners’ needs [62].

When providing feedback, our instructors ensure that their feedback includes components that help the learner improve, rather than providing feedback that is vague or unhelpful. For example, our feedback tries to follow an established pattern where the instructor observes the learner’s performance, provides advice, and compares his assessment with the learner’s own assessment [63]. We allow our learners to reflect on the feedback during the process, and the feedback is provided in a safe, non-judgemental learning environment. Based on the experience of others, we aim to provide feedback that is specific and timely [64, 65], describes task performance [66–68], and incorporates the learner’s goals and anticipated outcomes [69]. Other learners in the group are also invited to supply feedback.

### Assessing the Performance

After the learners have had a chance to improve their performance with feedback and reflection, they must then demonstrate this skill from start to finish, on the manikin, without interruption. Our goal is not to assess final competence as this relies on more formal standard setting methods. Rather, our purpose is to determine whether it is appropriate to allow the learner to perform the procedure on patients with ongoing supervision. Here, competence is not a one-time achievement; instead, it is a process or what Leach [70] refers to as a “habit” of life-long learning, and the learner needs to continue to demonstrate the procedure in the clinical context with supervision [71, 72].

Learners must demonstrate a checklist of components created by our faculty who have expertise in the procedure. If they satisfy the items in the proper sequence, they have satisfied our curricular standards for the procedure and can go on to perform the procedure, with supervision, in the clinical setting. Learners who fail to demonstrate the skills in the checklist redo the individual components, with feedback and reflection, before trying again.

### Enhancing Retention and Transfer

In this instructional event, retention refers to the learner’s ability to repeat the skill in future settings and transfer refers to the learner’s ability to adapt these skills to different situations [9].

Much of the groundwork for this instructional event will have been established by the preceding instructional events. For example, retention will be enhanced when active learning occurs while presenting the stimulus material and providing learning guidance. Retention is also enhanced when the learner’s own self-appraisal is incorporated while providing feedback, and the learner’s educational goals are accommodated while reviewing the learning objectives [55, 73, 74].

At this time, we administer the post-test and compare their performance to their pretest to reinforce what they have learned. Furthermore, we give the learners access to the demonstrational video to review in the future before performing the procedure again. This blended learning allows them to review the procedure in the proper clinical context, enhancing retention [75].

To promote transfer of knowledge and skills, we discuss how to modify the procedural technique in a variety of situations, such as when the patient has limited mobility and cannot maneuver into the proper position. We also try to instill an attitude of what Fraser and Greenlaugh [76] refer to as “capability”—that clinicians must adapt to situations which are novel and with which they may feel some uncertainty [77–79].

## Program Evaluation

Our program evaluation supports ongoing use of this instructional method. Before structuring our teaching, learners were asked whether the educational material was presented effectively and whether they felt they had acquired adequate procedural skills. Responses ranged from 2 (Disagree) to 5 (Strongly Agree) on a Likert scale from 1 to 5 for these two questions. After implementing our structured teaching, subsequent cohorts of learners consistently reported scores of 4 (Agree) and 5 for both questions. As well, we began to assess the learners’ procedural performance on an objective structured clinical examination (OSCE) station. On global ratings of performance—with possible scores from 1 to 5 and a score of “3” or higher needed to pass the station—all learners have received scores between 3 and 5.

## Conclusion

Using an instructional design model to craft components of the curriculum enables educators to structure the teaching so that all learners have a comparable learning experience. This structure, in turn, helps identify the specific program components that are effective and those that require improvement. While this article uses Gagne’s theory of instruction, there are many other educational paradigms that could be used for procedural instruction, such as Mayer’s instruction based on cognitive load theory [80], Peyton’s 4 step approach to procedural instruction [81], and Merrill’s First Principles of Instruction [82], to name a few.

Also, while this article has used one procedural skill as an example, an instructional design model can be used to program instruction for a variety of cognitive and procedural skills. Further study is needed to assess the effect that implementing an instructional design model into a teaching program has on workplace (that is, clinical) performance, such as the effect on procedural complication rates or speed.
